# Prevalence of Antibodies against Adeno-Associated Viruses (AAVs) in Göttingen Minipigs and Its Implications for Gene Therapy and Xenotransplantation

**DOI:** 10.3390/v16101613

**Published:** 2024-10-15

**Authors:** Kirsten Rosenmay Jacobsen, Javier Mota, Michelle Salerno, Alexis Willis, Dennis Pitts, Joachim Denner

**Affiliations:** 1 Ellegaard Göttingen Minipigs A/S, 4261 Dalmose, Denmark; 2 VRL Diagnostics, San Antonio, TX 78229, USA; javier.mota@vrl.net (J.M.); dennis.pitts@vrl.net (D.P.); 3 Marshall BioResources, North Rose, NY 14516, USA; msalerno@marshallbio.com (M.S.); awillis@marshallbio.com (A.W.); 4 Institute of Virology, Free University, 14163 Berlin, Germany

**Keywords:** adeno-associated viruses (AAVs), seroprevalence, gene therapy, xenotransplantation, Göttingen minipigs

## Abstract

Adeno-associated viruses (AAV) are widely used as delivery vectors in clinical trials for in vivo gene therapy due to their unique features. Göttingen minipigs are a well-established animal model for several diseases and can be used for the efficacy and safety testing of AAV-based gene therapy. Pre-existing antibodies against AAV may influence the results of testing and, therefore, the animals should be tested for the presence of antibodies against relevant AAV serotypes. The detection of AAVs in pigs may be also important for the virus safety of xenotransplantation. In this study, we screened Göttingen minipigs from Ellegaard Göttingen Minipigs A/S, Denmark, and Marshall BioResources, USA, for antibodies against AAV1, AAV2, AAV6, AAV9 serotypes. Of the 20 animals tested, 18 had no neutralizing antibodies for all AAVs tested, none had antibodies against AAV9, only one had antibodies against AAV6, and the titers of antibodies against AAV1 and AAV2 were less than 1:100, with two exceptions. For total binding IgG, more individuals showed positivity for all the tested serotypes but, in general, the levels were low or zero. Three animals had no antibodies at all against the AAVs tested. Therefore, Göttingen minipigs could be considered an attractive animal model for gene therapy studies. Since some animals were negative for all AAVs tested, these may be selected and used as donor animals for xenotransplantation.

## 1. Introduction

Adeno-associated viruses (AAVs) are small (26 nm in diameter) replication-defective, non-enveloped viruses with a linear single-stranded DNA (ssDNA) genome of 4.8 kilobases (kb). They belong to the genus *Dependoparvovirus*, which in turn belongs to the family *Parvoviridae* [1]. The genome encodes for viral replication, packaging, and capsid assembly proteins, these coding genes are flanked by two inverted terminal repeats (ITRs) [2]. There are at least 13 different AAV subtypes which have different tissue tropisms due to differing interactions with their receptor [3]. In 2012, the first gene therapy product, Glybera, which is based on an AAV gene-delivery approach for the treatment of patients with lipoprotein lipase deficiency, was approved in Europe and, today, AAV vectors are well established in clinical trials for in vivo gene therapy [4]. In 2019, 288 AAV-based clinical trials were registered with the US FDA database [5], and this number has increased rapidly until now [4]. Recently, it was shown that AAV-mediated gene replacement therapy was able to effectively rescue elements associated with the photoreceptor degeneration in Leber congenital amaurosis subtype 4 (LCA4) animal and cell models [6]. The AAV vector has unique features that are beneficial in clinical applications, including broad tropism, low immunogenicity, a relative ease of production, being non-pathogenic, rarely integrating into the host chromosome, and resulting in long-term expression of the transgene. AAVs are commonly used due to their high efficacy of transduction, high safety profile, and ease of production [4].

Naturally occurring AAVs infect both dividing and non-dividing cells in humans and animals and can remain latent in the host in a circular DNA episomal form; although integration in the host can occur, it is very infrequent. However, infection with wild-type AAVs could result in the production of neutralizing antibodies (NAbs), and these can interfere with treatment based on recombinant AAV vectors.

The Göttingen minipigs are an attractive animal model in many clinical areas due to their high similarity in anatomy, physiology, and immunology to humans [7,8,9]. Although considered as a large animal model, the Göttingen minipigs are smaller in size compared to other pig breeds, making them more manageable in a laboratory setting. Their cardiovascular, respiratory, digestive, and renal systems, as well as their metabolic functions, are closer to those of humans compared to other animal models. Anatomical similarities are important in studies that involve surgical procedures, medical device testing, or the development of surgical techniques. Göttingen minipigs can be used to model human diseases such as cardiovascular diseases, diabetes, obesity, and metabolic disorders. Because of the similarities of the porcine and human cardiovascular systems, Göttingen minipigs are widely used as a large animal model for cardiovascular diseases [10,11,12]. This allows for the preclinical testing of new therapies and interventions, including gene therapy studies.

Additionally, Göttingen minipigs are bred under controlled conditions, offering a high reproducibility when it comes to experimental data and results. As is the case for many other animal models used for AAV-based gene-delivery studies, knowing the AAV seroprevalence in Göttingen minipigs is highly relevant when considering the selection of animals with lower or no titers of pre-existing antibodies against AAV capsids. For example, in a study by Rapti et al. [13], the prevalence of antibodies against several AAV serotypes was assessed in a variety of small and large animal models (excluding non-human primates (NHPs)). The antibody titers varied widely between species and between serotypes. Rats displayed the lowest level of AAV NAbs, but they were present in all tested species (mice, rats, rabbits, dogs, sheep, and pigs) [13]. When Göttingen minipigs were injected with an AAV1 vector containing the coding sequence of the sarcoplasmatic reticulum ATPase (SERCA2a), the results showed that the vector genome copies in the heart were less prevalent in the animals with pre-existing neutralizing antibodies 2 days post-injection, demonstrating that even low NAbs against AAV vectors can have a negative transduction effect in vivo [12]. The wide prevalence of cross-reactive antibodies against AAVs in different animal species, including pigs, and wild-type AAVs that have been isolated from pigs [13,14,15,16] provide a potential explanation for the presence of neutralizing antibodies in the sera of minipigs.

In another study, three strains of pig (Norsvin Topigs-20 strain, Yucatan minipigs, and Göttingen minipigs) were used to assess the pre-existing antibodies against several AAV capsids, including AAV1, AAV2, AAV5, AAV6, AAV8, and AAV9 [17]. Although the results for the three strains cannot be compared because of the intrinsic strain differences, as well as the different ages used in this study, overall, the results showed a low prevalence of antibodies against most of the serotypes, except for AAV2. For example, in the Norsvin Topigs-20 strain, the seroprevalence of antibodies against AAV2 was very high. Interestingly, when the titers were defined in the positive animals, the Göttingen minipigs showed the lower titer (less than 1:20), while Topigs-20 and Yucatan minipigs had titers of 1:80–1:320 and 1:160–1:640, respectively. This study demonstrated that the minipig strains used in the current study could be used as animal models for gene therapy studies when using the AAV1, AAV5, AAV8, and AAV9 serotypes. Because of the high seropositivity against AAV2, as indicated above, and against AAV6, these two capsids are not recommended by the authors of this report for gene therapy studies. A prevalence of antibodies against multiple serotypes was also demonstrated, suggesting that there had been exposure to multiple viruses or that the pre-existing antibodies cross-reacted with multiple serotypes. These results demonstrate the usefulness of prescreening for pre-existing anti-AAV neutralizing antibodies.

When comparing Göttingen minipigs from Ellegard Göttingen Minipigs A/S with an isolated subpopulation of Göttingen minipigs which remained at the University of Göttingen, we found differences in the prevalence of porcine viruses. Whereas very few animals at Ellegard were infected with a porcine cytomegalovirus, which is actually a porcineroseolovirus (PCMV/PRV), hepatitis E virus (HEV), or porcine lymphotropic herpesvirus (PLHV), none of the animals at the Göttingen university were infected with these viruses [18]. It would be interesting to analyze whether there are also differences in the prevalence of AAVs.

Finally, AAV pre-screening should be considered not only in carrying out gene therapy studies but also in preparation for other common uses of Göttingen minipigs as animal models in biomedical research. In addition to gene therapy, knowledge of AAVs in Göttingen minipigs may be relevant for xenotransplantation, in which the patient typically undergoes significant immunosuppression. Göttingen minipigs have been considered for use as donor animals for islet cells in order to treat diabetic patients [19]. Therefore, these animals have previously been carefully screened for different xenotransplantation-relevant viruses [18,19,20,21,22,23,24,25]. AAVs were not screened for until now in these Göttingen minipigs. Most importantly, AAVs are not included in the list of viruses that are “not permitted in swine with designated pathogen-free status” that are to be used in xenotransplantation published by Fishman [26]. However, porcine adenovirus (pAdV) was included in this list. Although AAVs were thought to be non-pathogenic until recently, new data indicate an association of infection with AAV2 in children with acute hepatitis [27,28,29,30,31]. However, it is unclear whether AAVs can be transmitted, and whether they pose a risk for xenotransplantation. Here, we analyzed the Göttingen minipigs breed at Ellegaard Göttingen Minipigs in Denmark and at Marshall BioResources in the USA to measure the neutralizing and binding (total IgG) antibodies against four AAV serotypes.

## 2. Materials and Methods

### 2.1. Animals: Characterization and Housing

Göttingen minipigs between 3 and 6 months of age, males and females, were sampled. Animals were housed and sampled at the two breeding sites Marshall BioResources (MB, North Rose, NY, USA), supplying Göttingen minipigs to the North American market, and Ellegaard Göttingen Minipigs A/S (EGM, Dalmose, Denmark), supplying Göttingen minipigs mainly to the European market. Both facilities are AAALAC accredited.

Animals were housed in groups with non-study animals and selected from different pens and housing rooms. A total of 10 animals, 5 males and 5 females, were randomly selected from the following 4 barriers: EGM barriers 2 and 3 and MB barriers P1 and P3. The barriers are physically isolated from each other, and the animal colonies have been separated by multiple generations with no introduction of new biological material or animals.

### 2.2. Serum

Blood was sampled by placing the minipigs in a sling or at a V-bench and collected by vein puncture of the jugular vein. Serum tubes were left for 1 h at room temperature and centrifuged for 10 min at 1300× *g*. Serum was aliquoted into Eppendorf tubes in 0.5 mL aliquots and, thereafter, stored at from −70 °C to −80 °C until it was shipped to VRL Diagnostics on dry ice.

### 2.3. Testing for Neutralizing Antibodies (NAb)

To detect neutralizing antibodies (NAbs) against AAVs, we used a cell-based assay. Briefly, a HEK-293-derived cell line (which is proprietary and was modified to enhance AAV transduction efficiency) was grown and expanded using standard cell culture techniques. Cells were maintained in a CO_2_ incubator at 37 °C, using minimum essential medium (Corning MEM, Thermo Fisher Scientific Co., Waltham, MA, USA), and were supplemented with 1× sodium pyruvate, 1× penicillin and streptomycin mix, 1× non-essential amino acids, and 10% heat-inactivated FBS (Gibco, Thermo Fisher Scientific Co.). The day before the assay, cells were counted and seeded in 96-well plates (Corning, Life Sciences Inc., St. Petersburg, FL, USA) at a density of 25,000 cells per well, then they were incubated overnight as described above. On day 2, the serum samples were heat-inactivated at 56 °C for 30 min, then two-fold serially diluted (10 dilutions starting at 1:10) and incubated at 37 °C for 1 h in the presence of the correspondent AAV reporter virus (at the optimal MOI, see below) in a final volume of 50 μL of the same cell medium. The reporter viruses AAV1, AAV2, AAV6, and AAV9 (VectorBuilder Inc., Chicago, IL, USA) encode for the green fluorescent protein (GFP) used for the transduction efficiency measurement. The MOI for each virus (genome copies [Gc]) is 115, 40, 620, and 2300 for AAV1, AAV2, AAV6, and AAV9, respectively. The MOI for each virus was optimized as described previously [32] and applied for each cell transduction. Briefly, the permissible cells were seeded as described above and transduced with increasing MOIs (two-fold serial dilutions) of each AAV reporter virus; the transduction efficiencies were measured (GFP expression) at different time points and the optimal MOI was determined when a minimum of 90% of the cells showed GFP expression. Negative and positive controls were included in each plate. The negative control was a pool of pig serum samples that all individually tested negative for AAV seroreactivity, and the positive control was a pool of positive samples which all tested positive. The negative control served to evaluate the background, while the positive sera control confirmed that virus infection could be inhibited. Equal volumes of each sample were added to the pool, and small aliquots were taken and stored at −80 °C. From these pools, random aliquots were tested again to confirm positivity or negativity before use in the NAbs and total antibodies (TAbs) or binding antibodies assays. After incubation, the serum/reporter virus mix was added to the cells (with at least 50% confluency on day 2) in duplicate and incubated for several days in the CO_2_/37 °C incubator. Transduction levels of the GFP were assessed in a SpectraMax plate reader (Molecular Devices Co., Triangle, Wokingham, UK). The 50% inhibition cut-off was calculated based on the GFP signal from each sample and the maximum signal (cells plus AAV without serum), as well as the ‘no virus control’ (cells only). Results are reported as the specific serum titer in which the 50% inhibition occurs for each sample.

### 2.4. Testing for Total Antibodies (TAbs)

An indirect ELISA was developed and optimized to detect IgG antibodies against AAV viruses. Ninety-six-well plates (Corning, Life Sciences Inc.) were coated with virus-like particles (VLPs) corresponding to the AAV1, AAV2, AAV6, and AAV9 serotypes (VectorBuilder Inc., Chicago, IL, USA), at a concentration that varied from 0.5 to 2 μg/mL in coating buffer (Sera Care Inc., Gaithersburg, MD, USA), and incubated for 1 hr at room temperature (RT). After washing the plates, they were blocked with 2% BSA (Sigma Aldrich Co., St. Louis, MO, USA) for 2 hrs at RT. After the blocking step, serum samples (diluted 1:100 in in blocking solution) and appropriated negative and positive controls (described above) were added to the plate in duplicate and incubated for one hour at RT. Following the washing steps, a secondary anti-swine IgG, HRP conjugated antibody (Sera Care Inc.), was added at 1:2000 dilution (1% BSA-PBS) and incubated for 1 hr at RT. The plates were then washed and the SureBlue Reserve TMB 1-Component substrate (Sera Care Inc.) was added; after an incubation of 1–5 min, the reaction was stopped using TMB stop solution. The plates were immediately read for absorbance in a SpectraMax i3x plate reader (Molecular Devices, LLC, San Jose, CA, USA) at 450/630 nm. IgG titers were calculated by interpolating the sample OD value using a standard curve created with two-fold serial dilutions of a monoclonal antibody (proprietary), values were specific to each AAV serotype.

### 2.5. Statistical Analysis

**Figure 1:**
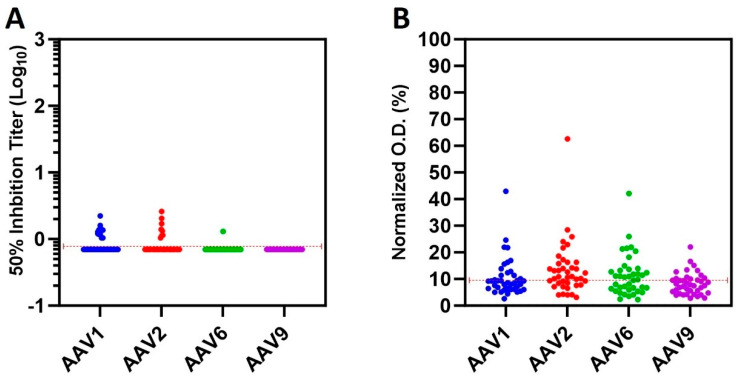
Analysis of pre-existing antibodies against several AAV serotypes. Neutralizing antibodies (NAb) were measured using a cell-based assay to determine the 50% transduction inhibition (**A**). For total IgG antibodies (TAb), samples were evaluated using an ELISA-based assay (**B**). The serotypes tested were AAV1, AAV2, AAV6, and AAV9 (indicated in the X-axis). The red dot line indicates the normalized 50% inhibition and the optical density (OD) cut-offs for the NAb and TAb, respectively.

Spearman r statistical test was used to evaluate any correlatuin between the NAb and TAb results. The data shown in Table 1 are graphically represented in Figure 1. Both plots and the Spearman test (for the correlation) were produced using Prism Version 10.3.1 (GraphPad software Inc., San Diego, CA, USA).

### 2.6. Ethics Statement

At both Marshall BioResources and Ellegaard Göttingen Minipigs, the samples were collected specifically for this study. Only one sample was taken from each animal. The samples were collected by trained staff. All experimental procedures were approved by an institutional and/or licensing committee. At Marshall BioResources, blood was sampled according to internal Institutional Animal Care and Use Committee (IACUC)-approved standard blood collection Standard Operating Procedure (SOP) for Blood Collection—All Species; USDA granted A-License, Certificate number 21-A-0008. At Ellegaard Göttingen Minipigs, blood sampling was covered by animal license granted by the Danish Animal Experiments Inspectorate, Ministry of Food, Agriculture and Fisheries in Denmark (license number 2022-15-0201-01167).

Marshall BioResources and Ellegaard Göttingen Minipigs are accredited by AAALAC International and fully comply with national regulations and the AAALAC’s Primary Standards, including the Guide for the Care and Use of Agricultural Animals in Research and Teaching (Ag Guide) and the Guide for the Care and Use of Laboratory Animals (NRC 2011). All methods are reported in accordance with ARRIVE guidelines (https://arriveguidelines.org, accessed on 8 October 2024).

### 2.7. Availability of Data and Materials

The datasets generated and analyzed during the current study are all available in this manuscript.

## 3. Results

### 3.1. Detection of Antibodies Neutralizing AAV in Göttingen Minipigs Sera

Göttingen minipigs from Ellegaard Göttingen Minipigs and from Marshall BioResources (20 animals from each facility) were screened using a cell-based neutralization assay for neutralizing antibodies (NAbs) that are specific to AAV1, AAV2, AAV6, and AAV9 (Table 1). With one exception, animal number 7 from the Ellegard Göttingen Minipigs Barrier 3, all animals had no or very low titers against all tested AAV serotypes.

### 3.2. Detection of Total Antibodies (TAbs) against AAV in Göttingen Minipigs Sera

When the same animals were tested for binding (total IgG) antibodies, it became clear that many animals were at some point in contact with different AAVs, or that cross-reacting antibodies were present in the serum (Table 2). Among the samples from Ellegaard Göttingen Minipigs, 8 out of 20 (40%) were infected with AAV1, 10 of 20 (50%) with AAV2, 11 of 20 (55%) with AAV6, and 5 of 20 (25%) with AAV9. Among the samples from Marshall BioResources, 14 of 20 (70%) were infected with AAV1, 18 of 20 (90%) with AAV2, 11 of 20 (55%) with AAV6, and 10 of 20 (50%) with AAV9. In both facilities, AAV2 had the highest prevalence (28 animals of 40, 70%), followed by AAV1 and AAV6 (22 of 40 animals, 55%, for both). AAV9 had the lowest prevalence (15 animals of 40, 37.5%). It is important to underline that there were numerous animals which were negative for antibodies against one or even all of the tested AAVs. Our TAb results demonstrate the low seroprevalence in the Göttingen minipigs.

### 3.3. Comparison of the NAbs and TAbs

As expected, while the number of samples in the NAb assay is low, the samples showing total binding IgG in the TAb assay are more abundant (Figure 1). It is important to note that both the NAb titer and the total concentration of anti-AAV IgG are low, and further analysis using simple linear regression (Spearman r statistical correlation test) showed that there is no correlation between the neutralizing antibodies and the total antibodies (The raw data for the Spearman test is provided in the Supplementary Table S1). The data indicate that, although more samples have antibodies that are able to bind to the virus particles, the number of samples with antibodies binding to regions of the virus required for infection of the target cells, and therefore neutralizing the virus, is lower. It is important to stress that some animals have neither neutralizing nor binding antibodies.

## 4. Discussion

This is the first systematic investigation of AAV-specific NAbs and TAbs in Göttingen minipigs in both production facilities, Ellegaard Göttingen Minipigs A/S, Denmark, and Marshall BioResources, USA. Our results show that the titers of NAbs are absent or, in general, low. NAbs are able to prevent infection, and they are usually directed against the receptor-binding site [2,33,34]. In contrast to the low prevalence of the NAbs in Göttingen minipigs, the number of samples with total virus-specific antibodies (total virus-specific IgG, also called binding antibodies) measured by the TAb assay was higher, although the concentration of the IgG was still low. The TAb assay detects both NAbs and non-neutralizing antibodies (nNAbs) [35], and the nNAbs bind to the virus but are unable to prevent the infection of the target cells. In order to find out if the amount of TAbs was correlated with the amount of NAb, we analyzed the presence of NAbs and total IgG using a Spearman r statistical correlation test, but we could not find any correlation (see Table S1). Although the size of the sample tested in this study was small, and more testing will be required, the low prevalences and the lack of correlation between the NAb and TAb results indicate that the naïve status of the Göttingen minipigs could be an important factor in considering this an amenable animal model for the use of AAV vectors. To add more support to the use of the Göttingen minipigs, other minipig strains have been used to evaluate AAV-based gene therapies, for example to evaluate liver-mediated gene expression [36]. In this study, although the TAb levels were not evaluated, the Nabs being present in low quantities allowed for the expression of the transgene in the target tissue. In another study, it was clearly shown that the prevalence of Nabs against six AAVs was lower in Göttingen minipigs compared with that in Norsvin Topigs-20 and Yucatan pigs [17]. The results were consistent with those obtained with NHPs, making this pig strain useful for gene therapy [36]. It is important to note that the seroprevalences reported here are lower compared to the seroprevalence found in non-human primates (NHPs) [16]. This supports the use of the Göttingen minipigs for gene therapy studies on AAV vectors. The differences in the titers of antibodies suggests that the prescreening of circulating anti-AAV antibodies could be helpful before the inclusion of pigs into studies. This will help to identify animals with no neutralizing antibodies against the tested variants of AAVs, e.g., AAV1, AAV2, AAV6, and AAV9. Gene therapy experiments using these vectors can thus be performed without problems in the virus-negative animals. Choosing a specific serotype for gene therapy depends on several factors such as the specific target, and others. For the purpose of this study, we chose four serotypes as a representative example, because they are the most commonly used serotypes, as reported in the literature. This selection does not indicate a level of relevance or importance and additional testing using other serotypes might be necessary.

The fact that, in rare cases (animals 4, 6, 11, 13, 17, 18 of EGM barrier 2), neutralizing antibodies were found in the absence of binding antibodies can be explained by cross-reacting antibodies against a different capsid, as reported previously [37]; however, further investigation is necessary to support this hypothesis.

As mentioned above, Dai at el. [17] also showed lower titers of neutralizing antibodies in Göttingen minipigs in comparison with other minipig breeds such as the Norsvin Topigs-20 strain and Yucatan minipigs. They also confirmed that AAV2 is the most common AAV in minipigs. Dai et al. [17] analyzed only Göttingen minipigs from Marshall BioResources, USA, whereas, in this study, we also analyzed animals from Ellegaard Göttingen Minipigs A/S, Denmark. It is interesting to compare these data (Table 3). In all previously studied Göttingen minipigs, Topigs-20, and Yucatan pigs [17], and the Marshall pigs studied here, AAV2 was the most common virus, whereas, in Göttingen minipigs from Ellegard Göttingen Minipigs, AAV1 was the most common virus. AAV9 was not found in all pigs in both studies. The differences in the prevalence of AAV1, AAV2, and AAV6 indicate changes in the virus load in the Göttingen Minipig breeds over time, in most cases a reduction of the prevalence, which is a positive development.

At present, using AAVs as a vector is the safest and most effective way to achieve gene delivery, and they are associated with long-term transgene expression [4]. The presence of pre-existing antibodies and the development of immune responses against the viral capsid post-dosing is one of the biggest obstacles when using AAVs for gene therapy. To address this, different strategies can be used, for example using modified capsids [29] or the use of capsids of a heterologous AAV. This is the case for the use of a porcine-derived capsid (AAV.Po.Guelph) that shows promising potential for use as a novel gene therapy vector [38]. However, special precautions should be taken when AAV capsids derived from animals are intended for use as delivery vectors because high seroreactivity has been reported, for example in the case of non-human primates (NHPs). Studies of seroprevalences against AAVrh10, a virus that originated in rhesus macaques, shows that about 59% of healthy human adults had antibodies against it, and 21% had NAbs [39]. On the other hand, AAV capsids may undergo recombination, which may be particularly advantageous. Recombination events in the cap gene may result in novel capsid characteristics, for example, (i) in the ability to bind to heparin sulfate proteoglycan; (ii) in the switching of epitopes from one recognized by pre-existing antibodies to one that the host might be naïve to; and (iii) in changes in the ability to bind receptors or co-receptors, thereby resulting in novel cell or tissue tropisms [39]. In some isolates of porcine AAVs, the capsid appeared to be the product of multiple recombination events between both porcine and human AAVs, increasing the effectivity of the AAV vectors [39]. Meanwhile, it has been shown that the use of low-endotoxin E. coli strain-derived plasmids reduces the AAV vector-mediated immune responses [40], a strategy which should be used in future trials.

It has been reported that most people have already been exposed to wild-type AAVs [16], resulting in the development of an immune response against them. These patients develop not only binding antibodies but also NAbs. These NAbs have the potential to neutralize the AAV vector, reducing its clinical efficacy. Furthermore, this complicates re-administration using the same capsid of AAV. Wang and collaborators [41] reported that the prevalence of pre-existing anti-capsid NAbs in the human population ranges from 40 to 74% for AAV8 and AAV2, respectively. Co-infection with different AAV serotypes and NAbs with cross-reactivity against other serotypes further complicates the situation. Therefore, simply switching to a different AAV serotype is not always a solution to increase the efficacy of gene therapy [16]. The seroprevalence of NAbs to AAV vector capsids may preclude a percentage of the human population from receiving gene therapy, especially if the gene therapy is to be applied systemically [42]. It is important to note that there are geographic differences in the prevalence of different AAVs in the human population. For example, the majority of healthy Chinese individuals were positive for NAbs, with the order AAV2 > AAV3 > AAV8 [43]. In a study analyzing 888 human serum samples from healthy volunteers in 10 countries around the world, neutralizing antibodies to AAV2 were the most prevalent antibodies in all regions, followed by antibodies to AAV1. The seroprevalences of antibodies to different AAVs was much higher in Belgium as compared with Italy [38].

Exposure to AAVs and the subsequent production of binding antibodies including NAbs was not only described for humans but also for other species used in preclinical studies such as pigs, dogs, and horses [13]. Therefore, it is important to evaluate large animal models for the presence of AAV NAbs before using them in gene therapy studies based on AAV vectors [14]. Furthermore, in rhesus monkeys, it was shown that the intramuscular injection of AAV vectors may be of advantage when circulating NAbs are present [3].

Interestingly, in a study analyzing the biodistribution of AAVs in pigs, the authors reported achieving an excellent transgene expression when using the AAV9 vector in cardiomyocytes, because intracoronary and intravenous delivery and a cardiomyocyte-specific promotor were applied. Although the AAV9 vector was also found in other tissues, including the liver, no toxic effects were observed and the vector was indeed successfully delivered to cardiomyocytes [44]. This and other studies [17,36] have demonstrated the presence of antibodies against AAVs in pigs, but have not analyzed the presence of NAbs and TAbs systematically as in the present study.

The presence of antibodies, both binding and neutralizing antibodies, when testing using human AAVs, indicates that the animals were infected with porcine AAVs, which induce cross-reacting antibodies against the human AAVs used for screening. This, however, does not mean that the virus is still present in the animal either as an episome or integrated into the genome of the animals, as it may have been eliminated by the immune system. This result also does not indicate whether other AAVs, in addition to the screened-for virus, are present in the animal. To get answers to this question, PCR-based methods using AAV-specific primers should be performed. When Bello et al. [45] isolated genomic DNA from pig gut and then screened for the presence of AAV sequences by using primers specific for conserved regions of the AAV genome, several new AAV sequences were isolated. However, these porcine AAVs had a high (60–80%) sequence homology to the human AAVs, explaining the presence of cross-reacting antibodies in the analyzed pigs.

It is common knowledge that the absence of antibodies against viruses indicates the absence of these viruses in the analyzed animals. Among the screened Göttingen minipigs, animals were identified which had no antibodies against AAV1, AAV2, AAV6, or AAV9. These animals can be selected by screening and these animals can be used for gene therapy trials. Furthermore, these animals also represent considerably safe animals for xenotransplantation. For a long time it was thought that AAVs are not pathogenic for humans [25], whereas some publications suggest a risk posed by these viruses. AAV2 sequences have been found in children with a non-A non-E acute hepatitis in the United States as well as in Scotland and in 34 other countries [28,29,30]. This virus was most likely acquired as a co-infection with human adenovirus, which is usually required as a ‘helper virus’ to support AAV2 replication [28]. Using next-generation sequencing (NGS), reverse transcription-polymerase chain reaction (RT-PCR), serology, and in situ hybridization (ISH), an infection with AAV2 in the plasma and liver samples of 26/32 (81%) hepatitis cases was observed and the virus AAV2 was detected within ballooned hepatocytes in liver biopsies [28].

The absence of viruses in the donor pigs which may be transmitted to the organs of the recipient and cause a disease, zoonosis, is important for xenotransplantation. It was shown that PCMV/PRV, when transmitted with a pig organ to non-human primates, was the cause of a significant reduction in the survival time of the animals [46]. Furthermore, recently, PCMV/PRV was transmitted to the first patient who received a pig heart and contributed to the early death of the patient [47]. Whether AAVs are present in donor pigs, and whether they can be transmitted and induce a disease, is still unknown.

Göttingen minipigs were thought to be used as donors of islet cells for the treatment of diabetes, and a preclinical trial in cynomolgus monkeys has been performed [19]. Therefore, these animals were analyzed extensively concerning the presence of pig viruses. However, Göttingen minipigs are not suited for the transplantation of organs due to their small size [48]. Göttingen minipigs from Ellegaard Göttingen Minipigs A/S, Denmark, have been screened for, altogether, 88 individual microorganisms, including bacteria, viruses, worms, fungi, and protozoa [21,22]. The following viruses were detected: HEV in 9 of 40 cases (22.5%), PCMV/PRV in 10 of 22 cases (45%), and PCV2 in 3 of 21 cases (14%); PCV3, PLHV-1, and PLHV-2 were not detected. Animals have been identified which were free of all viruses that were screened for. As expected, all 40 animals tested had PERV-A and PERV-B, but they also contained PERV-C in their genome. With the exception of PERV, all viruses, including AAV, can be eliminated by the selection of virus-negative animals, so that, finally, animals may be used for islet cell xenotransplantation which are free of all studied viruses. As shown here, AAV-negative animals exist and can be selected.

## 5. Conclusions

Göttingen minipigs from two commercial breeding facilities, Ellegaard Göttingen Minipigs A/S in Denmark, and Marshall BioResources in the USA, were screened for neutralizing and total antibodies against AAV1, AAV2, AAV6, and AAV9 serotypes. The animals had low or zero antibodies against these AAVs and, therefore, can be used as a larger animal model for gene therapy studies. Since animals exist which are free from all tested AAVs, AAV-negative animals can be selected, which may be advantageous for gene therapy and xenotransplantation.

## Figures and Tables

**Table 1:** Analysis of neutralizing antibodies against AAV1, AAV2, AAV6, and AAV9 in Göttingen minipigs. To evaluate the pre-existing antibodies against AAV viruses, serum samples were used in a cell-based neutralization assay. The specific titer at which the 50% transduction inhibition occurs is indicated. Samples from two different sources were used in this study: EGM, Ellegaard Göttingen Minipigs; MB, Marshall BioResources. Brown, positive above 1:10; blue, positive above 1:100.

Nr.	Sex	Age (Months)	Site	AAV1	AAV 2	AAV6	AAV9
1	Male	4	EGM-Barrier 2	<1:10	<1:10	<1:10	<1:10
2	Male	4	EGM-Barrier 2	1:40	1:21	<1:10	<1:10
3	Male	5	EGM-Barrier 2	1:19	<1:10	<1:10	<1:10
4	Male	5	EGM-Barrier 2	1:15	<1:10	<1:10	<1:10
5	Male	6	EGM-Barrier 2	1:10	<1:10	<1:10	<1:10
6	Male	4	EGM-Barrier 3	<1:10	<1:10	<1:10	<1:10
7	Male	5	EGM-Barrier 3	1:169	1:14	1:20	<1:10
8	Male	4	EGM-Barrier 3	<1:10	<1:10	<1:10	<1:10
9	Male	4	EGM-Barrier 3	<1:10	<1:10	<1:10	<1:10
10	Male	3	EGM-Barrier 3	1:25	1:25	<1:10	<1:10
11	Female	3	EGM-Barrier 2	1:28	<1:10	<1:10	<1:10
12	Female	5	EGM-Barrier 2	<1:10	<1:10	<1:10	<1:10
13	Female	3	EGM-Barrier 2	1:11	1:13	<1:10	<1:10
14	Female	4	EGM-Barrier 2	<1:10	<1:10	<1:10	<1:10
15	Female	6	EGM-Barrier 2	<1:10	<1:10	<1:10	<1:10
16	Female	4	EGM-Barrier 3	<1:10	<1:10	<1:10	<1:10
17	Female	4	EGM-Barrier 3	1:16	<1:10	<1:10	<1:10
18	Female	5	EGM-Barrier 3	1:23	1:50	<1:10	<1:10
19	Female	3	EGM-Barrier 3	1:15	<1:10	<1:10	<1:10
20	Female	3	EGM-Barrier 3	<1:10	<1:10	<1:10	<1:10
21	Male	6	MB-Barrier P1	<1:10	<1:10	<1:10	<1:10
22	Male	6	MB-Barrier P1	<1:10	<1:10	<1:10	<1:10
23	Male	5	MB-Barrier P1	<1:10	<1:10	<1:10	<1:10
24	Male	4	MB-Barrier P1	<1:10	<1:10	<1:10	<1:10
25	Male	3	MB-Barrier P1	<1:10	<1:10	<1:10	<1:10
26	Male	5	MB-Barrier P3	<1:10	1:405	<1:10	<1:10
27	Male	4	MB-Barrier P3	<1:10	<1:10	<1:10	<1:10
28	Male	3	MB-Barrier P3	<1:10	<1:10	<1:10	<1:10
29	Male	3	MB-Barrier P3	1:24	<1:10	<1:10	<1:10
30	Male	3	MB-Barrier P3	<1:10	1:11	<1:10	<1:10
31	Female	6	MB-Barrier P1	<1:10	<1:10	<1:10	<1:10
32	Female	5	MB-Barrier P1	<1:10	<1:10	<1:10	<1:10
33	Female	4	MB-Barrier P1	<1:10	<1:10	<1:10	<1:10
34	Female	3	MB-Barrier P1	<1:10	1:10	<1:10	<1:10
35	Female	3	MB-Barrier P1	<1:10	<1:10	<1:10	<1:10
36	Female	6	MB-Barrier P3	<1:10	<1:10	<1:10	<1:10
37	Female	5	MB-Barrier P3	<1:10	<1:10	<1:10	<1:10
38	Female	4	MB-Barrier P3	<1:10	1:111	<1:10	<1:10
39	Female	4	MB-Barrier P3	1:11	<1:10	<1:10	<1:10
40	Female	3	MB-Barrier P3	<1:10	<1:10	<1:10	<1:10

**Table 2:** Evaluation of total IgG against adeno-associated viruses (AAVs). To determine the presence of pre-existing antibodies against AAV1, AAV2, AAV6, and AAV9, total IgG was detected by an indirect ELISA assay (TAb assay). Two different sources of samples used in this study were: EGM, Ellegaard Göttingen Minipigs; MB, Marshall BioResources. Values shown are expressed in ug/mL; a negative value indicates below the lower limit of detection (LLOD). The LLOD for AAV1 was 0.15 µg/mL, for AAV2 it was 0.18 µg/mL, for AAV6 it was 0.22 µg/mL, and for AAV9 it was 0.15 µg/mL. Brown, positive above LLOD; blue, positive above 1.0; green, positive above 10.0.

Nr.	Sex	Age in Months	Site	AAV 1	AAV 2	AAV6	AAV9
1	Male	4	EGM-Barrier 2	Negative	Negative	Negative	Negative
2	Male	4	EGM-Barrier 2	0.25201	Negative	Negative	Negative
3	Male	5	EGM-Barrier 2	0.776246	0.930816	33.34828	0.57567
4	Male	5	EGM-Barrier 2	Negative	0.491444	Negative	Negative
5	Male	6	EGM-Barrier 2	0.317682	0.627378	23.2614	0.425245
6	Male	4	EGM-Barrier 3	Negative	Negative	0.541316	Negative
7	Male	5	EGM-Barrier 3	0.814247	Negative	0.581995	Negative
8	Male	4	EGM-Barrier 3	Negative	Negative	0.363293	Negative
9	Male	4	EGM-Barrier 3	Negative	1.362907	Negative	0.592366
10	Male	3	EGM-Barrier 3	0.426976	Negative	0.357067	Negative
11	Female	3	EGM-Barrier 2	Negative	0.461095	Negative	Negative
12	Female	5	EGM-Barrier 2	Negative	1.299849	Negative	1.064883
13	Female	3	EGM-Barrier 2	Negative	Negative	Negative	0.387974
14	Female	4	EGM-Barrier 2	0.305818	0.428783	Negative	Negative
15	Female	6	EGM-Barrier 2	0.287716	Negative	Negative	Negative
16	Female	4	EGM-Barrier 3	Negative	Negative	0.293477	Negative
17	Female	4	EGM-Barrier 3	Negative	0.740252	0.740252	Negative
18	Female	5	EGM-Barrier 3	Negative	0.853764	0.853764	Negative
19	Female	3	EGM-Barrier 3	0.585662	0.872661	0.872661	Negative
20	Female	3	EGM-Barrier 3	Negative	Negative	0.357512	Negative
21	Male	6	MB-Barrier P1	Negative	1.183842	16.7426	Negative
22	Male	6	MB-Barrier P1	0.897421	1.932243	22.51668	0.536595
23	Male	5	MB-Barrier P1	0.247912	1.602681	Negative	Negative
24	Male	4	MB-Barrier P1	0.290711	0.62899	Negative	Negative
25	Male	3	MB-Barrier P1	0.25009	0.45749	Negative	Negative
26	Male	5	MB-Barrier P3	Negative	14.84969	Negative	0.389731
27	Male	4	MB-Barrier P3	0.36085	0.551178	Negative	0.743084
28	Male	3	MB-Barrier P3	Negative	Negative	Negative	Negative
29	Male	3	MB-Barrier P3	0.570132	0.740142	25.45707	0.417732
30	Male	3	MB-Barrier P3	1.868564	0.596776	148.296	0.476848
31	Female	6	MB-Barrier P1	0.269211	0.651258	17.63796	0.382716
32	Female	5	MB-Barrier P1	Negative	Negative	Negative	Negative
33	Female	4	MB-Barrier P1	0.295997	0.595387	17.20962	Negative
34	Female	3	MB-Barrier P1	Negative	0.449497	Negative	Negative
35	Female	3	MB-Barrier P1	0.360078	0.570097	39.48218	0.353615
36	Female	6	MB-Barrier P3	0.45102	0.769629	21.69672	0.440174
37	Female	5	MB-Barrier P3	0.293442	1.403329	18.9477	0.408475
38	Female	4	MB-Barrier P3	0.517462	0.834601	43.73907	0.660524
39	Female	4	MB-Barrier P3	0.413928	0.636124	19.98774	Negative
40	Female	3	MB-Barrier P3	Negative	0.424109	Negative	Negative

**Table 3:** Prevalence of AAV in Göttingen minipigs—a comparative analysis.

AAV	Marshall BioResources, USA		Ellegaard Göttingen Minipigs A/S, Denmark
	Dai et al. [17] n = 22	This Study n = 20	This Study n = 20
AAV1	23%	10%	55%
AAV2	83%	15%	25%
AAV5	9%	n.t.	n.t.
AAV6	55%	0%	5%
AAV8	5%	n.t.	n.t.
AAV9	0%	0%	0%

n.t., not tested.

## Data Availability

All data are available in this manuscript.

## Supplementary Materials

The following supporting information can be downloaded at: https://www.mdpi.com/article/10.3390/v16101613/s1, Table S1: Raw data for the NAb and TAb analysis.
