# Supplementary material for: Prevalence of Antibodies against Adeno-Associated Viruses (AAVs) in Göttingen Minipigs and Its Implications for Gene Therapy and Xenotransplantation
Source: Viruses. 2024 Oct 15;16(10):1613. doi: 10.3390/v16101613 (PMC11512330; doi:10.3390/v16101613)
Supplement: Supplementary file 1 [file viruses-16-01613-s001.zip › viruses-3209106-supplementary.pdf]

Table S1. Raw data for the NAb and TAb analysis presented in Figure 1 and Table 1. NAb data is the 50% inhibition titer transformed to Log10, and the TAb data is the Optical Density (O.D.) value transformed to percentage (%).

	NAb data Normalized (Log)					TAb normalized data (%)				
Animal ID	AAV1	AAV 2	AAV6	AAV9		Animal ID	AAV1	AAV 2	AAV6	AAV9
**245920**	0.69897	0.69897	0.69897	0.69897		**245920**	9.197222	8.45122	8.099715	7.529954
**245953**	0.69897	0.69897	0.69897	0.69897		**245953**	10.10278	13.78354	13.93732	10.38479
**246115**	1.278754	0.69897	0.69897	0.69897		**246115**	21.93333	18.62805	18.21652	13.4977
**246160**	1.176091	0.69897	0.69897	0.69897		**246160**	5.102778	10.94207	4.992877	8.423963
**246166**	0.69897	0.69897	0.69897	0.69897		**246166**	6.427778	22.98476	2.283476	22.07834
**246355**	0.69897	0.69897	0.69897	0.69897		**246355**	9.747222	9.405488	5.420228	4.440092
**246377**	0.69897	0.69897	0.69897	0.69897		**246377**	2.588889	4.262195	3.574074	3.960829
**246451**	1.60206	1.322219	0.69897	0.69897		**246451**	8.083333	7.792683	7.212251	8.18894
**246563**	1.447158	0.69897	0.69897	0.69897		**246563**	7.247222	10.21951	6.991453	6.882488
**246686**	1.041393	1.113943	0.69897	0.69897		**246686**	6.469444	8.54878	6.713675	9.564516
**354654**	1.361728	1.69897	0.69897	0.69897		**354654**	9.411111	10.60671	12.70085	9.347926
**354852**	2.227887	1.146128	1.30103	0.69897		**354852**	21.75	7.094512	21.51852	4.064516
**355000**	0.69897	0.69897	0.69897	0.69897		**355000**	7.602778	3.146341	9.754986	2.764977
**355047**	1.20412	0.69897	0.69897	0.69897		**355047**	9.272222	9.179878	11.06268	4.175115
**355121**	0.69897	0.69897	0.69897	0.69897		**355121**	8.363889	6.542683	11.47721	4.025346
**355283**	0.69897	0.69897	0.69897	0.69897		**355283**	5.497222	4.091463	9.558405	3.449309
**355287**	0.69897	0.69897	0.69897	0.69897		**355287**	5.088889	16.29878	8.04416	13.24885
**355324**	1.176091	0.69897	0.69897	0.69897		**355324**	16.3	10.83841	21.25499	9.085253
**355446**	1.39794	1.39794	0.69897	0.69897		**355446**	12.38056	4.006098	25.9416	4.202765
**355464**	0.69897	0.69897	0.69897	0.69897		**355464**	6.861111	4.012195	11.75499	2.873272
**1223551**	0.69897	0.69897	0.69897	0.69897		**1223551**	6.013889	21.74085	10.69231	5.285714
**1223691**	0.69897	0.69897	0.69897	0.69897		**1223691**	24.64722	28.47561	13.58832	12.71659
**1223747**	0.69897	0.69897	0.69897	0.69897		**1223747**	8.625	14.22866	11.16524	9.447005
**1226428**	0.69897	0.69897	0.69897	0.69897		**1226428**	7.952778	25.84146	4.236467	6.762673
**1226649**	0.69897	0.69897	0.69897	0.69897		**1226649**	6.016667	7.155488	4.754986	5.665899
**1228684**	0.69897	0.69897	0.69897	0.69897		**1228684**	9.288889	13.81402	5.381766	7.891705
**1229257**	0.69897	0.69897	0.69897	0.69897		**1229257**	9.45	13.16463	10.94017	7.476959
**1229966**	0.69897	0.69897	0.69897	0.69897		**1229966**	8.022222	10.1311	6.367521	5.59447
**1230018**	0.69897	0.69897	0.69897	0.69897		**1230018**	6.180556	9.932927	5.944444	5.930876
**1231529**	0.69897	0.69897	0.69897	0.69897		**1231529**	11.34444	12.65549	20.48006	8.788018
**3153908**	0.69897	0.69897	0.69897	0.69897		**3153908**	13.87778	16.25305	13.19801	10.70737
**3155196**	0.69897	2.607455	0.69897	0.69897		**3155196**	5.072222	62.63415	4.084046	9.603687
**3155293**	0.69897	0.69897	0.69897	0.69897		**3155293**	9.372222	24.01829	11.84046	10.01843
**3158993**	0.69897	0.69897	0.69897	0.69897		**3157695**	15.63611	17.2561	21.92877	15.13594
**3159752**	0.69897	0.69897	0.69897	0.69897		**3158551**	12.86389	13.94817	12.36325	7.223502
**3160220**	1.380211	0.69897	0.69897	0.69897		**3158993**	11.36667	12.2622	6.860399	16.66129
**3160289**	0.69897	1.041393	0.69897	0.69897		**3159418**	4.433333	9.283537	2.48433	4.829493
**3157695**	0.69897	2.045323	0.69897	0.69897		**3159752**	5.880556	7.588415	6.680912	6.269585
**3158551**	1.041393	0.69897	0.69897	0.69897		**3160220**	16.98333	15.77439	14.93732	10.2212
**3159418**	0.69897	0.69897	0.69897	0.69897		**3160289**	42.91944	13.19207	42.11538	11.48618
